# The American Medical Association—Thirty-Third Annual Session, Saint Paul, Minnesota, June 6–9, 1882

**Published:** 1882-07

**Authors:** 


### Societies.

### THE AMERICAN MEDICAL ASSOCIATION—THIRTY-THIRD ANNUAL SESSION—SAINT PAUL, MINNESOTA, JUNE 6–9, 1882.

*For the Atlanta Medical Register.*

The Association was called to order at the appointed hour by Dr. A. J. Stone, chairman of the committee of arrangements, and the exercises were opened with prayer by Right Rev. Bishop Ireland. Governor Lucius F. Hubbard was then introduced, and he delivered the formal address of welcome. The Governor, in appropriate words of cordial greeting, invited the gentlemen present to the full enjoyment of the attractions which he thought the city and the State offered, and extolling the virtues of the climate, he said: "There is a characteristic of our country of peculiar interest to, and that invites the investigation of gentlemen of your profession. The exhilarating and vitalizing influences of our climate have made Minnesota the Mecca for health seekers in years past, and as a direct result of your visit at this time, we shall expect to greatly widen and strengthen our reputation in that regard. With assurances of health that do not disappoint, and opportunities for the acquisition of wealth that can but satisfy, we invite the brain and muscle of the world to a home in our midst."

At the conclusion of these remarks, which were greeted with hearty applause, the Ex-Presidents and Ex-Vice Presidents of the Association, who were present, were invited to seats on the platform. Drs. Sayre, Davis, Toner, and Cole responded and the appearance of each one, as he stepped forward, was the signal for renewed applause.

The secretary announced the reception of protests from several State and local societies, against the admission of delegates from the medical society of the State of New York, on account of the recent action of said society in connection with the code of medical ethics. He also read a letter from Dr. Samuel D. Gross, expressing regret at his necessary absence, and denouncing in vigorous language the action of the New York society, as an insult to the Association as a body and also to the individual members thereof. These papers were referred to the judicial council.

Dr. Stone, in behalf of the committee of arrangements, said, when you receive cards of regulation you will find an invitation to a reception at the Metropolitan hotel and receptions at private residences. On these occasions all members are expected to be accompanied by a lady or ladies. For so large an association St. Paul has but limited hotel accommodations, but the hearts of the people are large, and it is only necessary for any one to report his inability to find proper accommodation to be properly accommodated. The members of the committee of arrangements wear badges to designate their position, and each member will feel aggrieved if he is not put to all the trouble possible in looking after such and kindred matters. Thursday afternoon you are invited to a banquet and sail on Lake St. Croix, by the Hon. D. M. Sabin, of Stillwater. The Chicago, St. Paul and Omaha road has placed a train at your disposal, of which every one of you will be a conductor, so you can go and come to suit your own convenience. There is but one regular excursion tendered, that is by the Lake Superior Transportation company, for a sail upon one of the palatial steamers of the company on that magnificent sheet of water.

At this point Dr. P. O. Hooper, of Arkansas, first vice-president and acting president of the Association, was introduced and he proceeded to deliver the annual address. In opening Dr. Hooper fittingly feeling said:

"A letter received some weeks ago, from our distinguished president, Dr. J. J. Woodward, conveyed to me the sad intelligence that he would be unable to meet with us on this occasion, by reason of physical infirmity. In foreign lands he seeks to repair his health, wasted by arduous labor and exhausting vigils, and to rebuild his frame, shattered by a harrassing accident. We all feel a keen disappointment in the absence of one so eminent, whose gifted intellect and matured experience have been acknowledged and appreciated, not only by those who have been intimately associated with him in the army, but by all who have enjoyed his fascinating society, or possessed a share of his warm friendship. May the airs he has sought bring healing on their wings, and speedily return him to the welcoming shores of his native land, completely restored to the full measure of a man's strength and vigor."

The speaker then briefly reviewed the history and remarked the progress and the useful, scientific work of the Association, as attested by the thirty-two ponderous volumes of transactions which like mile stones indicate its line of march since its formation. He referred favorably to the establishment and regular issue of a journal in lieu of the publication of the proceedings in book form. He congratulated the association upon the influence it had exerted upon national legislation and claimed the national board of health as one of the many fruits of the labor of the body. He deplored the extensive prevalence of a preventable disease—small-pox—in this country, and expressed the belief that proper attention to the application of the great prophylactic—vaccination—would surely prevent its periodic visitations. Its existence amongst us is due he thought not alone to neglect on the part of the people of preventive means, but also to carelessness and inattention on the part of medical men themselves. He advocated the adoption of some plan looking to the relief of impoverished, disabled and helpless members of the medical profession and to the aid of their defenseless widows and orphans. The speaker closed his eloquent address with a beautiful tribute to the memory of his immediate predecessor in the office which he now fills, the late Dr. Hogden, of Saint Louis, and made an earnest appeal to the younger men in the profession to emulate his bright example and to imitate his sterling virtues. Referring to the code of ethics he used the following incisive words:

"We have a code of medical ethics, the best ever given for the government of medical men, of acknowledged force and effect, of universal acceptance in every State of the Union ; and it is now too late for any single physician or State society to oppose or set at defiance the moral power of this body. Had the Association done nothing else than to originate and adopt these beautiful precepts, which should govern in our relations to each other, to our patients and to the public, it would have done a service entitling it to everlasting gratitude, and to an imperishable name in the annals of our country. There has recently been exhibited by a few a disposition to be restive under the operations of certain portions of the code. It may not, perhaps, be becoming in me to discuss this matter here, as it will be for judicial investigation and decision; but I may be permitted to suggest, that we should not retreat from our well-chosen lines of defense. One mistaken movement would involve us in a whirl of inconsistencies tending to place us in a false attitude and bring dishonor upon the profession. The broad lines of demarkation should never be obliterated. Our Association stands prominently forth in its high purposes, and its means of accomplishing these purposes are distinctly enunciated.

In the discussion of all ethical questions, a spirit of liberalism, has always mingled with a never sleeping sense of imperative obligation to the established truths of science, of order, of law. I do not say, that the time may never come, for there is no perfect work of mortal hands, when your organic laws will require modification, and amendment; but until the time does arrive, when the impulse of the great heart of the profession shall be felt, and radical changes be demanded in the light of a perfected knowledge, let us maintain without internal strife, the unsullied standard of professional honor and morals, now "full high advanced" in our midst, and decline association with those who will not recognize that flag, or who having once recognized, have abandoned it. We should, without reservation, declare the perpetuity of this Association. Changes have not been accepted unchallenged, but controversy has been enlightened and tolerant."

In reply to a letter from the President, Dr. J. J. Woodward, expressive of his disappointment and regret at his inability to attend the meeting, the secretary was instructed to send a cable-gram to him conveying the greeting and sympathy of the Association.

The following letter was read, and is printed as a contribution toward the code controversy:

No. 285 Fifth Avenue, New York, May 22, 1882.

J. Manlius Smith, M. D, Secretary New York State Medical Society—Dear Sir:—I have just received from you my certificate as delegate from the New York State Medical Society to the American Medical Association, to be held in St. Paul on the 6th of June. As the State society has *ignored* the code of ethics by which they were bound as members of the association, I can not see how they can expect their delegate to be received by an Association whose laws they refuse to obey, and which must, therefore, refuse them admittance. I therefore respectfully decline to act as its delegate, and hereby return my certificate.

Very respectfully, Lewis A. Sayre, M. D.

As illustrative of the cordial sincerity of the reception tendered the Association we reproduce the following invitation which was read on the morning of the second day, and remark, in passing, that it was one among many of like character which were received.

City of Fargo, Office City Clerk,
Fargo, D. T., June 5, 1882.

Whereas, The American Medical Association, including in its members many eminent scientists, scholars and benefactors from every portion of our own country, and representatives from other lands, assembles in annual session this week in our neighbor city of St. Paul:

*Resolved*, That we hereby extend to the members of this distinguished body a cordial and earnest invitation to visit our rising young city; see for themselves the beauty and prosperity of this garden spot of the new world, the Red River valley of Dakota, and accept the hospitality of our people, which is tendered them with open hearts and hands and houses. W. A. Kindred, Mayor.

Chas. P. Thompson, City Clerk.

The judicial council reported as follows:

In regard to the protest against the receiving of delegates from the New York State Medical Society, which was referred to us, the judicial council decide as follows:

Having carefully examined the code of ethics adopted by the New York State Medical Society at its annual meeting in February, 1882, as furnished us by the secretary of said society, the judicial council find in said code provisions essentially differing from and in conflict with the code of ethics of this Association, and therefore, in accordance with provision of rule 9 of the by-laws of this Association, decide unanimously that the said New York Society is not entitled to delegates in the American Medical Association.

This report was received with long continued applause.

Dr. W. F. Holt, of Macon, represented Georgia upon the committee on nominations.

The roster showed that the State of Georgia was represented at the meeting by Drs. Robert Battey, J. W. Bailey, A. W. Calhoun, E. L. Connally, H. F. Campbell, J. L. Hamilton, W. F. Holt, Jno. Thad. Johnson, J. G. Thomas, J. S. Todd and W. F. Westmoreland.

The committee on nominations made the following report, and its adoption elected the officers named therein:

The chairman of the committee, Dr. Foster Pratt, in submitting the report, stated that the action of the committee had been unanimous on every question.

President—John L. Atlee, Philadelphia.

First Vice-President—Eugene Grissom, North Carolina.

Second Vice-President—A. J. Stone, Minnesota.

Third Vice-President—J. A. Octerlony, Kentucky.

Fourth Vice-President—H. S. Orme, California.

Treasurer—J. R. Dunglison, Philadelphia.

Librarian—C. H. A. Kleinschmidt, Washington.

Chairman of committee on arrangements—X. C. Scott, Cleveland.

Assistant Secretary—I. N. Hines, Cleveland.

Members of Judicial Council—N. S. Davis, Illinois; J. M. Brown, United States navy; X. C. Scott, Ohio; M. Sexton, Indiana; N. C. Husted, New York; Wm. Lee, Maryland; J. E. Rives, West Virginia.

*Sections*—Practice of Medicine—J. H. Hollister, Illinois, chairman; J. G. Lee, Pennsylvania, secretary.

Surgery and Anatomy—W. F. Peck, Iowa, chairman; Paul F. Eve, Tennessee, secretary.

Obstetrics—J. K. Bartlett, Wisconsin, chairman; G. A. Moses, Missouri, secretary.

Medical Jurisprudence and State Medicine—Foster Pratt, Michigan, chairman; Thos. L. Neal, Ohio, secretary.

Ophthalmology, Otology and Laryngology—A. W. Calhoun, Georgia, chairman; Carl Seiler, Pennsylvania, secretary.

Diseases of Children—R. Blount, Indiana, chairman; J. H. Sears, Texas, secretary.

Dentistry—D. H. Goodwillie, New York, chairman; T. W. Brophy, Illinois, secretary.

*Committee on Necrology*—Dr. J. M. Toner, Washington, chairman; Alabama, vacant; California, Dr. J. H. Woolsey; Colorado, Dr. Charles Dennison; Connecticut, Dr. W. C. Burke; Dakota, Dr. O. S. Pine; Florida, vacant; Delaware, vacant; Georgia, Dr. Robert Battey; Illinois, Dr. J. H. Holister; Indiana, Dr. G. L. Sutton; Iowa, Dr. S. B. Chase; Kansas, Dr. C. V. Maleram; Kentucky, Dr. J. W. Brooks;

Louisiana, Dr. J. W. Dupree; Maine, Dr. A. Garcelon; Maryland, Dr. Wm. Lee; Massachusetts, Dr. H. O. Marcey; Michigan, Dr. W. F. Breakey; Minnesota, Dr. D. W. Hand; Mississippi, Dr. H. A. Grant; Missouri, Dr. C. L. Hall; Nebraska, Dr. R. C. Moore; New Hampshire, vacant; New York, Dr. J. A. Chapman; New Jersey, Dr. E. A. Watson; North Carolina, Dr. C. W. Woolen; Ohio, Dr. J. F. Russell; Oregon, vacant; Pennsylvania, Dr. Frank Woodbury; Rhode Island, Dr. A. Ballou; South Carolina, vacant; Tennessee, Dr. J. B. Lindsley; Texas, Dr. H. C. Ghent; Vermont, Dr. O. F. Tassett; Virginia, Dr. F. D. Cunningham; West Virginia, I. V. K. Curtis; Wisconsin, J. T. Reeve; United States Army, G. Perrin; United States Navy, F. M. Grinnell; United States Marine Hospital Service, F. W. Miller.

*Committee on Publication.*--Drs. W. B. Atkinson, Albert Fricke, J. S. Cohen, F. Woodbury, J. H. Packard, J. R. Dunglison, J. V. Shoemaker.

*Committee on State Medicine:*—Alabama, vacant; Arkansas, D. C. Ewing; California, J. O. Tucker; Colorado, C. Dennison; Connecticut, W. C. Willey; Delaware, vacant; District of Columbia, D. W. C. Patterson; Dakota, S. B. McGlumpt; Florida, vacant; Georgia, H. F. Campbell; Illinois, H. A. Johnson; Indiana, T. M. Stevens; Iowa, D. W. Crouse; Kansas, J. Bell; Kentucky, T. B. Greenly; Louisiana, J. W. Dupree; Maine, F. B. Ferguson; Maryland, Wm. Lee; Massachusetts, M. G. Parker; Michigan, R. C. Kedzie; Minnesota, C. N. Hewitt; Mississippi, H. A. Grant; Missouri, E. W. Schauffler; Nebraska, E. M. Whillen; New Hampshire, J. A. Sanborn; New Jersey, D. C. English; New York, E. M. Moore; North Carolina, C. W. Woollen; Ohio, J. Ransohoff; Oregon, vacant; Pennsylvania, A. H. Smith; Rhode Island, J. N. Eldridge; South Carolina, vacant; Tennessee, V. S. Lindsley; Texas, W. H. Parke; Virginia, F. D. Cunningham; Vermont, S. W. Thayer; West Virginia, John Frizzel; Wisconsin, J. T. Reeve; United States Navy, A. L. Gihon; United States Hospital Service, F. W. Miller.

The committee also announced that Cleveland, Ohio, had been selected as the place of meeting next year.

The special committee appointed at the last session of the Association to report upon the feasibility of substituting a weekly journal for the annual volume of transactions, and to present a plan indicating how it could best be accomplished, made an elaborate report favoring the proposition, the consideration of which was made the special order for the third day. When the question came up it was disposed of by the unanimous adoption of the following resolutions:

*Resolved,* That the interests of the Association would be promoted by the publication of its transactions in a weekly medical journal under its own control, instead of in an annual volume as heretofore, provided it could be done without involving pecuniary embarrassment, or so far engrossing its funds as to prevent the annual encouragement of original investigation by its members.

*Resolved,* That so much of the report of the committee on printing the transactions as relates to the increase of membership of this Association by application from members of state and local societies be and the same is hereby approved.

*Resolved,* That so much of the report of the committee on journalizing the transactions of the Association as relates to the appointment of a board of trustees, nine in number, and their duties, be and the same is hereby adopted, and that the president of the Association should appoint a special committee of seven to recommend to this meeting of the Association the names of nine members for election to constitute said board of trustees.

*Resolved,* That the board of trustees so appointed be requested to agree upon a plan of a medical journal, to be called the JOURNAL OF THE AMERICAN MEDICAL ASSOCIATION, and to send circulars explaining such plan, and asking pledges of support by actual subscriptions, to the members of the medical profession throughout the whole country, and thereby ascertain as reliably as possible what degree of support the proposed journal can have as a basis for commencing its publication; and that said board also proceed to ascertain and agree upon the best methods of publishing said journal, the best editorial services it can secure to take charge of the work, and the best plan for its issue.

*Resolved*, That said board of trustees be and are hereby instructed to retain under all circumstances, in whatever plans or contracts proposed for adoption, entire control over the advertising as well as other pages of the journal that is proposed to be established, and that said board report in full at the next meeting of this Association, the plans upon which it has been able to agree, together with the response of the profession to its circular asking actual subscriptions to the proposed journal, and that the constitutional amendments proposed by Dr. Packard last year be continued upon the table until the report of the board of trustees is received and acted upon.

*Resolved*, That the treasurer of this Association is hereby authorized to pay out of funds in the treasury the necessary expenses of the board of trustees in printing and distributing its circulars and in conducting its proper correspondence.

*Resolved*, That the committee of publication proceed to publish the proceedings and transactions of the present meeting in a volume as heretofore, using all diligence to give it an early distribution to those entitled to receive it.

Subsequently the president appointed the following-named delegates as a committee to appoint the board of trustees, in accordance with the resolution, viz:

Dr. L. A. Sayre, of New York.

Dr. J. M. Toner, of District of Columbia.

Dr. J. Foster Pratt, of Michigan.

Dr. J. R. Dunglison, of Pennsylvania.

Dr. Robert Battey, of Georgia.

Dr. W. J. Peck, of Iowa.

Dr. H. O. Marcey, of Massachusetts.

By Dr. A. L. Gihon, U. S. N.:

*Resolved*, That it is the sense of the American Medical Association that it will be conducive to justice and the dignity of the profession that medical expert testimony shall be given without having the appearance of being in behalf of either side, but to be stated simply as facts.

The resolution was adopted amid loud applause.

Dr. J. G. Thomas, Georgia, offered the following resolution:

*Resolved*, That the Association approve the organization of faculties in medicine having no other foundation than the examination for degrees as a measure which will increase the value of the present methods of education in medical colleges in this country.

After discussion on the resolution a motion to lay it on the table was lost. Ayes, 75—nays, 132.

The resolution was finally postponed by a unanimous vote.

Dr. Dennison, Colorado, offered the following resolution which was adopted:

Resolved, That no action of this Association, either in its code or its annual meetings, shall be construed to commit members of the American Medical Association to the adherence of any dogma, and members should have a care not to allow their names to be erroneusly registered as allopathists, etc., in State and city registration of physicians.

A motion was made to vote the usual amount to the secretary for his work, provided that amount was left in the treasury after defraying the expenses of the publication of the journal.

Dr. Toner thought that $500 was sufficient. Dr. Davis wanted some definite sum mentioned.

The motion to make the sum $500 was lost and $1,000 ordered paid the secretary subject to the condition of the treasury.

Dr. Toner gave notice of a charge in the constitution so as to make the secretary serve without compensation.

Dr. H. Goodwiley's proposed amendment to article 11, section 8, permanent members, strikes out the words "but without the right of voting."

Dr. Pratt of Michigan offered an amendment to article 13 of the by-laws, altering it so as to read that none but members present shall be eligible to the office of president, first vice-president, secretary, treasurer, chairman of sections and member of the judicial council.

Dr. Keller announced an amendment allowing the committee on nominations until a date as late as September after the annual meetings to select the place of meeting. Laid over under the rules for one year.

Dr. Keller, of Arkansas, moved that the secretaries of the State societies send annually to the secretary of the American Medical Association a list of the members dropped from the State societies. Adopted.

N. S. Davis introduced the following:

Resolved, That after the next annual meeting the permanent interests and influence of this Association would be best promoted by again holding every second meeting in Washington, as its home on common national ground and not as invited guests, while each alternate meeting should be held in such section of the Union as would be most useful in promoting the society organizations in all parts of our country. Adopted.

Dr. Sayre, as one of the committee to select trustees for the journal, said that the duty of the committee in selecting a board of trustees was a very important one. That in making the selection the committee had been pampered by the resolution passed on Thursday, to the effect that non-attending members were not eligible, as some of the oldest and ablest members of the Association were unable to be present at this session.

The trustees appointed by the committee were as follows:

For three years—Drs. Davis, Chicago; Moore, New York, and Toner, Washington.

For two years—Drs. Campbell, Georgia; Packard, Pennsylvania, and Connor, Michigan.

For one year—Drs. Hooper, Arkansas; Garcelon, Maine, and McMurty, Kentucky.

Subsequently at a meeting of the trustees, Dr. N. S. Davis was elected president and Dr. F. H. Packard, secretary.

On motion, Dr. Davis, Chicago, was appointed to escort Dr. John L. Atlee, the president elect to the platform. Dr. Atlee was greeted with loud applause, and on being introduced by President Hooper, made the follong address:

Gentlemen of the American Medical Association: "It is with no ordinary emotions that, by your partiality, I occupy a chair that I have seen filled by a Chapman, a Warner, a Stevens, a Knight and a host of worthies, living and dead, who were and are the ornaments of our profession. I beg you to accept, gentlemen, my heartfelt thanks for the honor you have conferred upon me. I accept it also with gratitude as a tribute to the memory of a dear brother, who, were he living, would more deservedly occupy this position. My chief motive in coming here on this occasion was to assist in carrying out the instructions unanimously given by the Lancaster County Medical Society to uphold the honor and dignity of our noble profession by putting the seal of condemnation upon the recent action of a State society; the sanction of which would have given character to a system of practice derogatory to common sense, and professional integrity. All honor, gentlemen, to the report of our judiciary committee. In the performance of my duties I shall endeavor to be firm and impartial and I trust that I may be supported by your kindness and courtesy in trying to uphold the right."

Space will not allow, in this issue, a synopsis of the many excellent papers read before the several sections. They will, however, be accessible to most of our readers in the volume of transactions, which will probably be issued this year more promptly than usual.

After the passage of numerous resolutions of thanks the Association adjourned, on the fourth day, to meet in Cleveland, Ohio, on the first Tuesday in June, 1883.

